# Imaging feature of cosmetic fillers in cone-beam computed tomography and its dental consideration

**DOI:** 10.1186/s13005-022-00327-0

**Published:** 2022-07-08

**Authors:** Chena Lee, Yoon Joo Choi, Kug Jin Jeon, Seong Ho Choi, Sang-Sun Han

**Affiliations:** 1 Department of Oral and Maxillofacial Radiology, Yonsei University College of Dentistry, 50-1 Yonsei-ro, Seodaemun-gu Seoul, Korea; 2 Department of Periodontology, Yonsei University College of Dentistry, Seoul, Korea

**Keywords:** Cone-beam computed tomography, Cosmetic filler, Differential diagnosis, Imaging diagnosis

## Abstract

**Background:**

As the application of cone-beam computed tomography (CBCT) in head and neck area increases for dental treatment purposes, cosmetic filler materials are incidentally observed. Since the materials are very diverse, unnecessary referrals or additional examination may be performed when clinicians are unfamiliar with the imaging findings. Thus, this study aimed to introduce the imaging characteristics of cosmetic fillers and grafts shown in dental CBCT with dental considerations that the clinician should be aware of.

**Methods:**

CBCT obtained for dental purpose presenting cosmetic material were selected. The location of the material was identified as buccal, retroantral, parotid space, nose, zygoma, and symphysis. The material was classified as single or multiple, and grouped according to morphology: speckle, round, eggshell, linear, and amorphous. The radiopacity was classified as similar to soft tissue, between soft and hard tissue, similar to hard tissue, and metal.

**Results:**

Twenty-one patients were reviewed, and all patients were female with mean age of 50.5 years. The buccal space was the most frequent location for multiple filler materials. The symphysis was the next frequent location and only single material were shown in this location. Cases having multiple filler showed diverse shapes while all single materials showed round shape. Fillers showing radiopacity of hard tissue were similar to diseases producing soft tissue calcifications. Metal-density material distributed in spaces induced white and dark streak artifacts in the CBCT image. All single materials presented radiopacity between soft and hard tissue and attached to the bone surface causing mandibular bone resorption.

**Conclusions:**

Cosmetic materials displayed various imaging features in CBCT acquired during dental procedure. Clinicians should consider that cosmetic material may cause mandibular bone resorption and imaging artifacts on CBCT. Knowledge of the imaging characteristics of cosmetic fillers may help correct diagnosis.

## Background

Since introduction of cone-beam computed tomography (CBCT) in dentistry, it has been rapidly expanded and applied in the field of dental procedures [1]. Nowadays, CBCT acquisition became an essential part in planning implant installation surgery. With substantial usage of CBCT in routine practices, there is a growing demand for education in interpretation of CBCT as well [2, 3]. Unexpected incidental findings in CBCT, which were not aware previously on intraoral or panoramic radiography, may distract clinicians and cause concern regarding the differential diagnosis and need for referral. Fillers and graft materials for cosmetic purpose shown in dental CBCT are one such imaging finding that may frustrate clinicians.

Currently, cosmetic filler materials have been developed rapidly and vary widely [4]. In addition, the frequency of facial filler injection has been increasing. These materials themselves are diverse and they may interact within body tissues and present more varied imaging characteristics [4–7]. It is more challenging for clinicians to deal with such findings. Thus, many reports have introduced imaging characteristics according to materials, mostly in multi-detector computed tomography, magnetic resonance imaging, and positron emission tomography [4, 6, 7]. However, studies on CBCT imaging are rare and difficult to find in the literature.

Gold thread for facial lifting that show high density have been reported, and many dentists recognize it as foreign material as they are presenting characteristic feature even on panoramic radiography [8]. Meanwhile, materials with relatively low density similar to soft and hard tissue may confuse clinicians whether they are disease or foreign body. If clinicians have knowledge of several specific patterns of cosmetic fillers in CBCT images, a differential diagnosis can be made by taking the patient’s previous history.

Therefore, in this study, the imaging characteristics of various cosmetic fillers and grafts on CBCT images are introduced to aid clinicians in making a differential diagnosis. In addition, possible filler material-related complications that should be considered during dental treatment are presented.

## Materials and methods

### Subjects

Patients who underwent panoramic radiography and CBCT examination for dental treatment purposes between March 2017 and April 2021 in dental hospital were reviewed. The CBCT images presenting any foreign material were selected and the patient’s filler injection history was thoroughly reviewed. Patient consent was waived by the IRB due to the retrospective nature of this study.

### Clinical and imaging analysis

Patients’ demographic information, age, sex, and history were reviewed. The CBCT imaging characteristics were analyzed according to the location, density, and numerical and morphological features of the materials. Panoramic radiography was also evaluated to determine whether the material was observed in the image.

#### Location

**Fig. 1:**
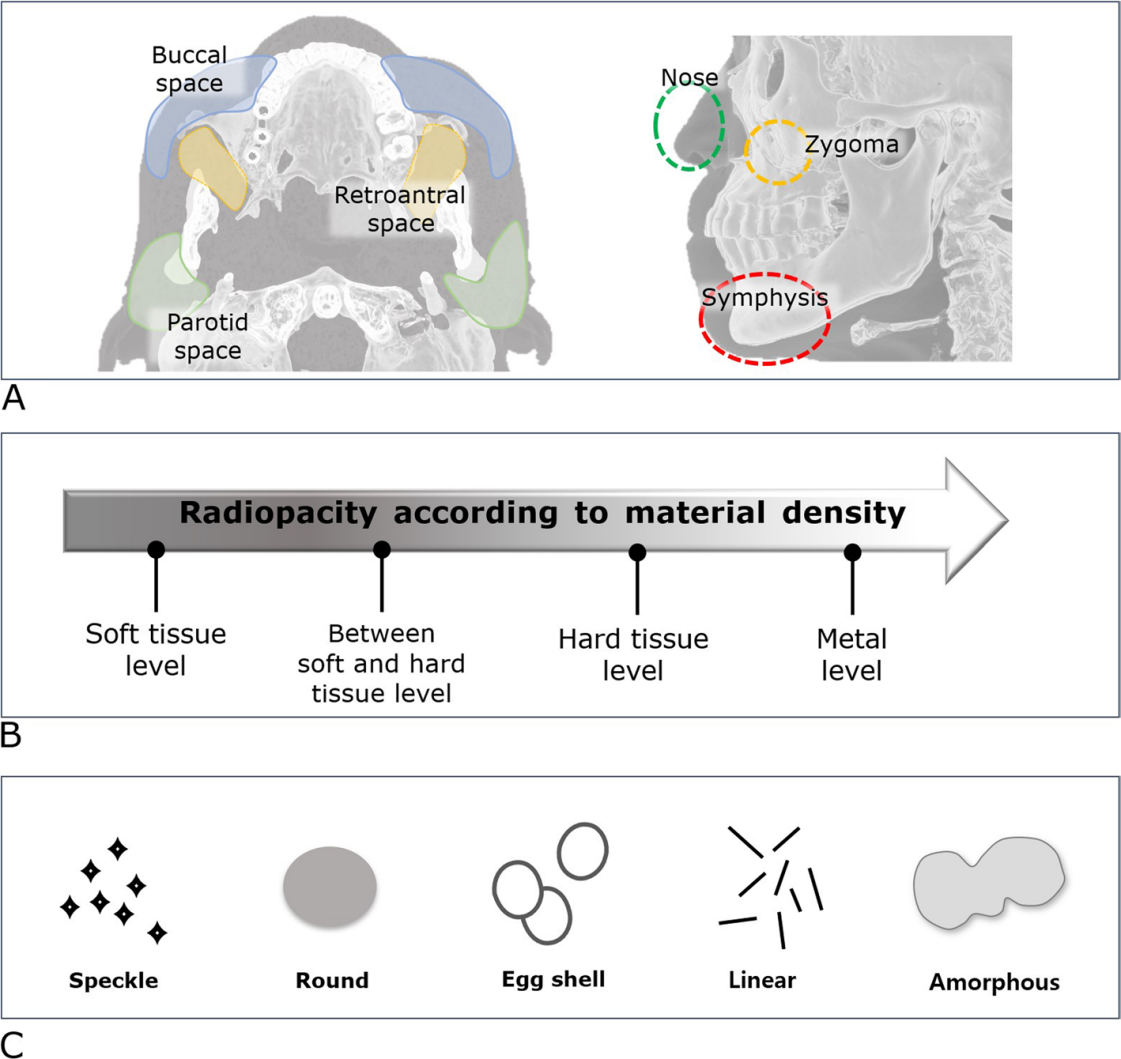
Cosmetic filler material classification criteria. **A** The anatomic location of the material. **B, C** The imaging characteristics consider radiopacity and morphology on cone-beam computed tomography

The location was classified as the buccal space, retroantral space, parotid space, dorsum of the nose, zygomatic process of the maxilla, and symphysis (Fig. 1A).

#### Density

According to the radiopacity on CBCT, the materials were divided into four different density groups: (1) similar to soft tissue, (2) soft and hard tissue, (3) similar to hard tissue, and (4) metal (Fig. 1B).

#### Morphological feature

The materials were also classified as single and multiple. Considering the shape of the fillers, the morphological features were classified into five different types: speckle, eggshell, linear, round, or amorphous (Fig. 1C).

## Results

In total, CBCT images from 22 patients were reviewed. All patients were female, and the age was ranged from 22 to 79 years with an average age of 50.5 ± 22.9 years. Table 1 presents the overall information of the patients according to the imaging characteristics of the material.

**Table 1:** Imaging and clinical characteristics of cosmetic filler and grafts

Morphology		Density	Panoramic radiography	Mean age (years)	Location	Number of cases
Single	Round	Between soft and hard tissue	Not visible	32.9 ± 13.8	Symphysis	6
					Zygoma	1
Multiple	Speckles	Hard tissue	Not visible	58.3 ± 22.8	Buccal space	2
					Buccal, retroantral, parotid space	1
	Eggshell and speckle mixture	Hard tissue	Partly visible	76.0 ± 3.6	Buccal space	2
					Buccal, retroantral space	1
	Round and eggshell mixture	Soft and hard tissue mixture	Not visible	64.5 ± 6.4	Buccal, retroantral space	2
	Round and amorphous mixtures	Soft tissue	Not visible	54.8 ± 17.3	Buccal space	4
	Linear	Metal	Visible	40.0 ± 24.0	Buccal space	1
					Buccal, parotid space	1
					Nose	1

**Fig. 2:**
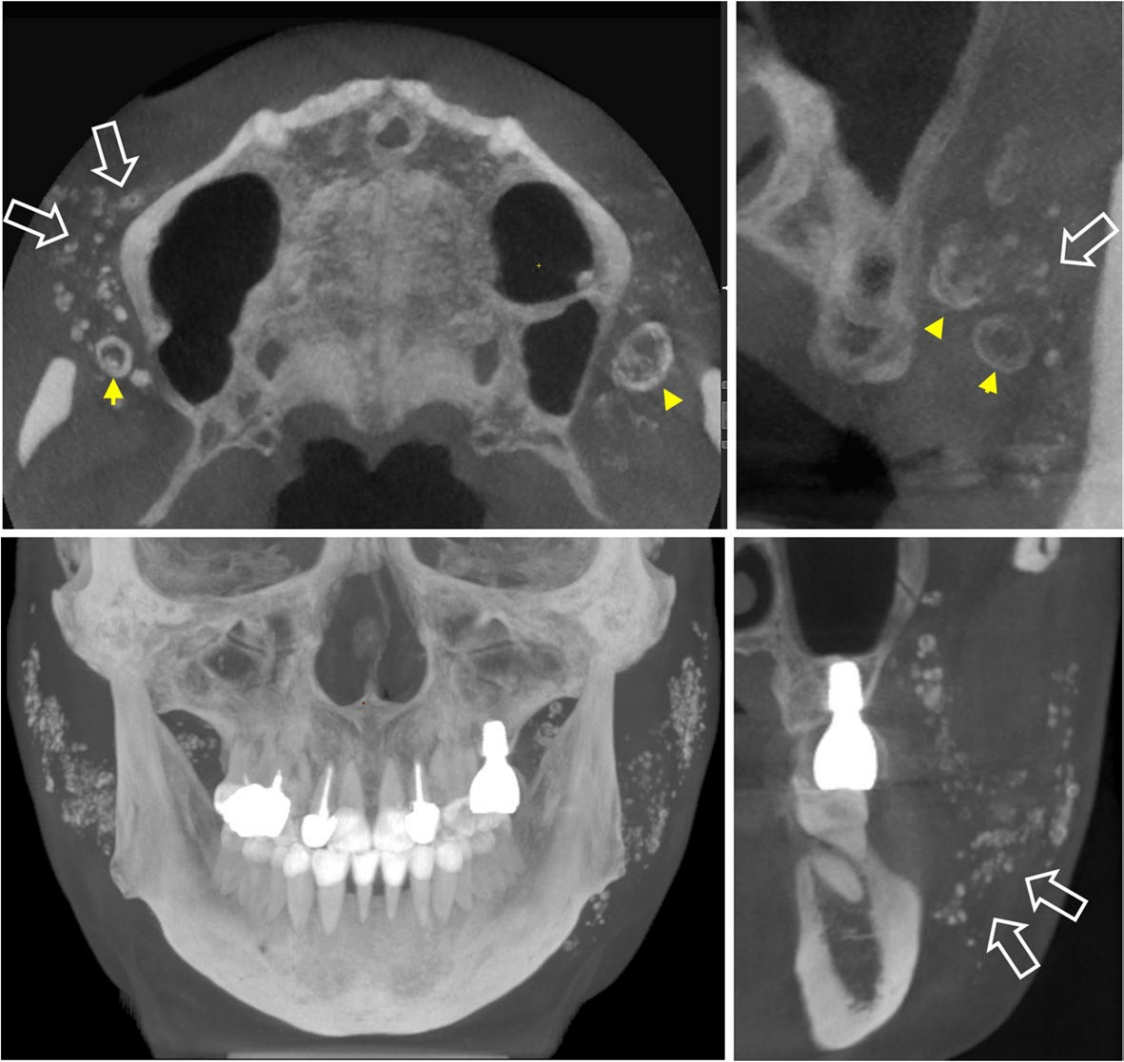
Multiple materials with radiopacity similar to that of hard tissue. Speckled fillers (hollow arrow). Eggshell-like filler (arrowhead)

**Fig. 3:**
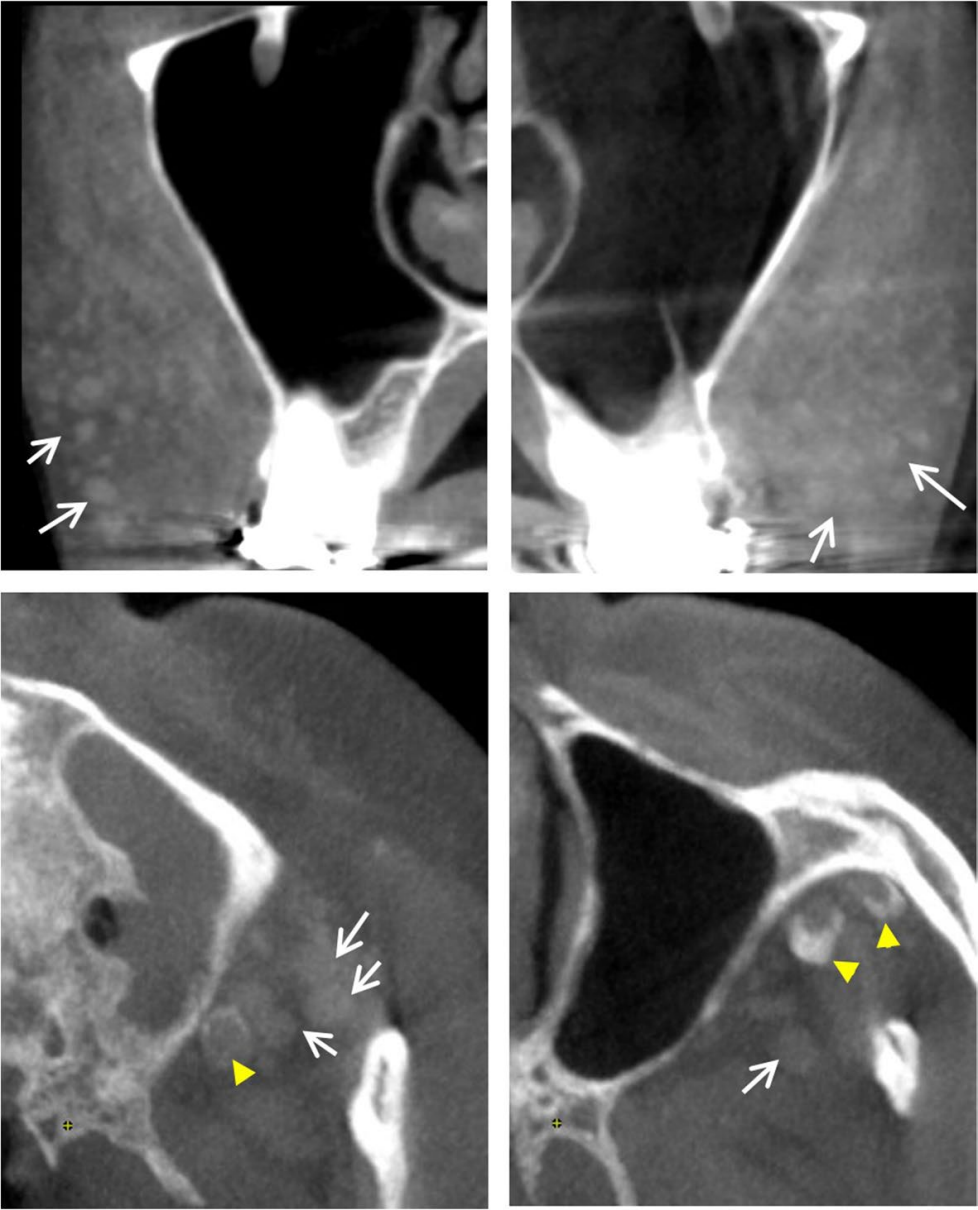
Multiple round and amorphous materials. Fillers showing radiopacity similar to that of soft tissue (white arrow). Fillers with radiopacity similar to that of soft tissue mixed with radiopacity similar to that of eggshell-like hard tissue (arrowhead)

**Fig. 4:**
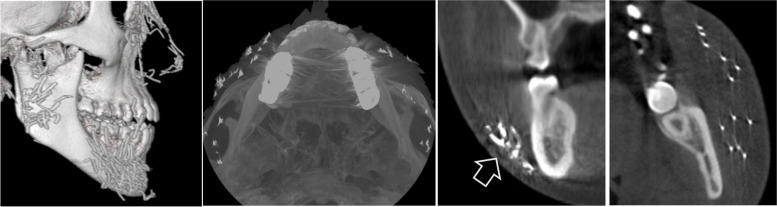
Multiple linear materials show high radiopacity comparable to metal (hollow arrow). Note that the material induces image artifacts

The buccal space was the most frequent location for cosmetic filler materials. Fourteen patients had material within the buccal space; among them, three patients had materials within both the buccal and retroantral spaces, and one patient showed buccal and parotid spaces filled with materials. One patient showed material distribution over the buccal, retroantral, and parotid spaces. These materials were all multiple and appeared as various combinations of density and morphology (Fig. 2, 3). Among them, eight patients presented materials with radiopacity similar to hard tissue mimicking pathology, such as phlebolith, miliary osteoma cutis, or lymph node calcification. Materials showing a metal-like density (*n* = 3) were linear in shape, and characteristically induced dark and white streak artifacts in CBCT images (Fig. 4).

**Fig. 5:**
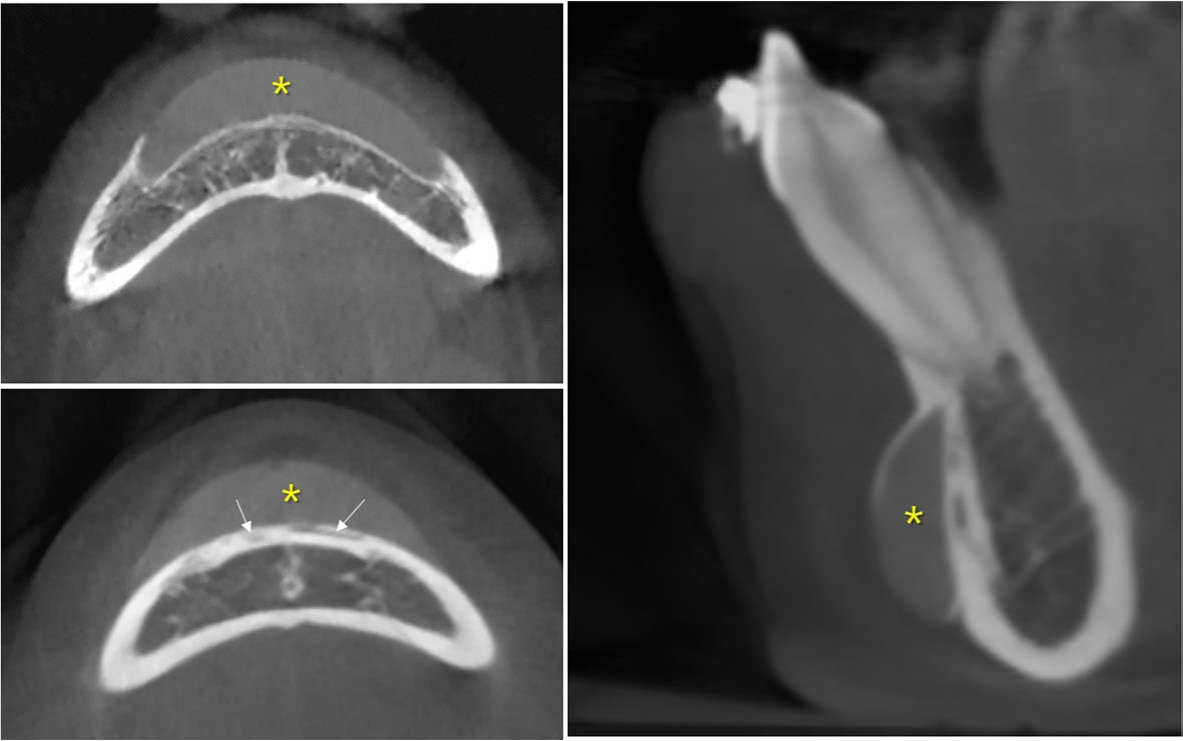
The graft material (asterisk) shows radiopacity between that of soft and hard tissue. The material has eroded the bone surface to which it is attached (arrow). Advanced bone resorption with little bone remaining between the tooth and the material

Seven patients showed a single round material with radiopacity between that of soft and hard tissue. The materials were mostly observed on the symphysis (*n* = 6); however, one patient had material on the zygomatic process (*n* = 1). All materials were directly attached to the bone surface and induced bone resorption (Fig. 5).

## Discussion

The introduction of CBCT allows dental clinicians to obtain more information than that obtained from two-dimensional radiography. Cosmetic fillers may confuse clinicians, as imaging characteristics in CBCT images have not yet been reported. Over the past few decades, various types of cosmetic fillers and grafts have been introduced, and the wide variability of their imaging characteristics requires differential diagnosis from pathologic conditions [4, 5].

In this study, the graft material in the symphysis area was associated with critical findings related to the maxillofacial bone. This type of implant accounted for seven out of the 21 cases in this study and could be misdiagnosed as a tumor due to the remodeling of the symphysis bone to which it was attached. For chin augmentation, polyethylene and silicone implants are the most popular materials and bone erosion is a common side effect of these implants [5, 9, 10]. Infection, hematoma, or migration of materials is another complication reported [5, 10]. In this study, one of the patients with symphysis augmentation showed upward migration of the graft and bone resorption. This caused material contact with the periapical region of the mandibular anterior teeth. The tendency of migration and bone erosion of this graft material may cause complications during dental treatment.

Meanwhile, for other cosmetic filler materials scattered in the facial spaces, there are no special considerations for dental surgery if they are correctly recognized. This pattern of materials, that is, multiple scattering patterns, is seen with fluid injectable silicone or paraffin wax, although the use of paraffin wax has been stopped after the 1980s [6]. Most injectable fluid cosmetic materials cannot be visualized on CBCT considering their density [11]. However, these injectable materials may cause a gradual inflammatory reaction and leading to the development of granulomas or dystrophic calcification, which can be observed on CBCT [12, 13].

Some of the calcified materials formed due to the inflammatory response to the material mimic phlebolith occurring with venous malformation. Moreover, venous malformations frequently occur in the retroantral space. When the filler material migrates into the retroantral space and forms an eggshell calcification, it is difficult to make a differential diagnosis [6]. Venous malformation frequently involves mucosa and adjacent bone structures, thus dental procedures may cause uncontrolled bleeding. For differential diagnosis, clinicians should remember that filler calcification appears bilaterally compared to phleboliths that mostly appear on one side. In addition, filler calcification mainly occurs in elderly female patients. For a definitive diagnosis, it will be helpful to confirm by asking the patient for an injection history.

Speckled or round filler calcification may be confused with miliary osteoma cutis or lymph node calcifications [6]. In miliary osteoma cutis, small calcification is dispersed in the cutaneous layer, while lymph node calcification commonly occurs due to a history of tuberculosis [14]. As a benign extraskeletal hard tissue formation [15], it does not cause any problems for dental treatment if there are no additional complications.

Two patients showed potential oral pathogenicity. The filler materials were dispersed in the anterior region of the parotid gland. Several previous studies have reported that injectable filler material obstructs the salivary duct and causes mucocele or sialadenitis [4, 16]. Therefore, if the patient complains of discomforts related with dry mouth or obstructive sialadenitis, and shows the imaging pattern of one of the filler materials described in this study, a careful examination should be performed. Removal of the filler material can be considered.

Only two patients presented metal-like materials within the facial spaces. As a high-density material, a gold thread is a known foreign material, which can be observed on panoramic radiography [8]. In this study, it was relatively easy to recognize metallic materials on CBCT because of the linear shape and radiopacity. In addition, these materials generated white and dark artifacts in the images. Because of the small size and thinness of the material, they did not produce massive artifacts masking the maxillofacial bone, but massive gold threads may cause overall poor image quality.

## Conclusions

Careful investigation of previous history and knowledge of the imaging characteristics of cosmetic fillers may help to make a correct diagnosis. In addition, clinicians should consider that some cosmetic graft materials may cause dental complications, including alveolar bone resorption and imaging artifacts, especially on CBCT.

## Data Availability

The datasets acquired during and/or analyzed during the current study are available from the corresponding author on reasonable request.

## Abbreviation

CBCT: Cone-beam computed tomography
